# Evaluation of Peanut Physiological Responses to Heat and Drought Stress Across Growth Chamber and Field Environments

**DOI:** 10.3390/plants14172687

**Published:** 2025-08-28

**Authors:** Ranadheer Reddy Vennam, Keely M. Beard, David C. Haak, Maria Balota

**Affiliations:** 1 School of Plant and Environmental Sciences, Virginia Tech, Blacksburg, VA 24061, USA; keelyb17@vt.edu (K.M.B.); dhaak@vt.edu (D.C.H.); 2 Tidewater Agricultural Research and Extension Center, Virginia Tech, Suffolk, VA 23437, USA

**Keywords:** combined heat and drought, growth chamber, stomatal conductance, stress progression, Virginia-type peanuts

## Abstract

Heat-exacerbated drought stress is becoming increasingly common in crop production systems, including peanuts, yet limited information exists on how peanut cultivars respond to this combined stress. While controlled environments allow for the isolation of these stress effects, their relevance to field conditions remains unclear. In this study, five Virginia-type peanut cultivars were evaluated under four treatments in a growth chamber environment, i.e., control, heat, drought, and combined heat and drought stress; and under two treatments in the field environment, i.e., rainfed control, and combined heat and drought stress using rainout shelters. The physiological traits assessed included stomatal conductance and transpiration rate, as well as leaf temperature difference. In both environments, combined heat and drought resulted in a significant decline in physiological performance compared to control conditions. On average, stomatal conductance decreased by 65% in the growth chamber and 21% in the field under combined heat and drought stress, while transpiration was reduced by 49% and 24%, respectively. In the growth chamber, leaf temperature difference increased by 40% under combined stress, whereas it was not statistically different under field conditions. Correlations of the physiological responses between growth chamber and field were stronger under combined stress conditions than under control conditions. Principal component analysis revealed clear genotypic separation based on gas exchange and thermal traits, with NC 20 and Sullivan consistently associated with higher stomatal conductance and transpiration under stress across environments, indicating greater physiological resilience, while Emery clustered with traits linked to stress susceptibility. These findings underscore the significant impacts of combined stress in peanut production and highlight the importance of evaluating cultivar responses under both controlled and field environments to guide crop improvement strategies.

## 1. Introduction

Crop production worldwide is increasingly being challenged by unfavorable weather, with heat and drought stress becoming more frequent, intense, and prolonged, including in peanut (*Arachis hypogaea* L.) producing regions [1,2,3]. While peanuts are a warm-season crop, they are susceptible to water stress caused by heat-exacerbated drought, especially during the flowering and pod-filling stages. Heat stress can reduce flower production and pollen viability, cause cellular membrane damage, and impair pod filling [4,5,6,7]. Similarly, drought can decrease stomatal conductance and transpiration rate, and increase oxidative damage, limiting photosynthesis, plant growth, and reproductive success [8]. In addition, drought can reduce nodule formation and biological nitrogen fixation [9] and lead to increased aflatoxin contamination [10].

Previous research from our group on peanuts has shown that drought stress negatively impacted relative chlorophyll content, specific leaf area, PS II photosystem efficiency, canopy temperature, stomatal conductance, transpiration, carbon assimilation, and yield [3,11,12,13]. Additionally, Singh et al. (2016) [7] found that heat stress increased membrane injury and reduced chlorophyll fluorescence, while Beard et al. (2023) [14] reported a decline in pollen viability due to elevated temperatures. In other crops, research has shown that combined heat and drought can have a more significant effect on plant physiological performance than each stress alone, including maize (*Zea mays* L.), soybean (*Glycine max* L.), wheat (*Triticum aestivum* L.), and rice (*Oryza sativa* L.) [15,16,17,18]. In peanuts, there is limited understanding of how the combination of heat and drought stresses may affect crop physiology and production [1,19,20]. In the Virginia-Carolina peanut belt of the United States, rainfed production is the most common practice, with irrigation availability on less than 15% of the peanut acreage [12]. This increases peanuts susceptibility to the co-occurrence of heat and drought during mid-season (July–August), which could lead to significant yield losses if stress-resilient genotypes are not developed [21].

Peanuts, with their above-ground flowering and below-ground pod development, are susceptible to deviations from the optimal soil moisture, temperature, and root zone conditions [22]. Understanding peanut responses to combined heat and drought is important to improving stress resilience. Stomatal conductance rate, transpiration rate, and leaf temperature are reliable indicators of plant water status, heat load, and stress tolerance. They are widely used in phenotyping to select resilient genotypes. For instance, based on stomatal conductance and transpiration rates, Zhang et al. (2022) [21] identified two distinct water-use strategies among several peanut genotypes in response to mid-season drought, i.e., “water-saving” and “water-spending” strategies. Interestingly, the study found high-yielding, drought-resilient genotypes within both strategy groups, suggesting that different physiological mechanisms can confer drought tolerance. Using leaf temperature and chlorophyll fluorescence in the field and controlled conditions, Singh et al. (2016, 2014b) [7,23] reported drought and heat-tolerant peanut genotypes. Santos et al. (2024) [24] demonstrated that water deficit priming, i.e., the application of a mild, controlled drought during early growth stages to physiologically pre-condition plants for future water stress has enhanced stress tolerance and water use efficiency in peanuts because priming allowed for expression of physiological adaptation to stress. These studies underscore the value of physiological screening in breeding for stress resilience in peanuts.

To dissect stress responses, researchers often utilize controlled environments, such as growth chambers and greenhouses, to precisely regulate microclimatic conditions, including temperature, relative humidity, and soil moisture [25]. These setups allow for repeatable experiments that isolate targeted stress factors. However, translating data from controlled conditions to open-field environments is complicated [25,26]. In field settings, crop performance is influenced by numerous interacting weather and soil variables, e.g., light intensity, wind, vapor pressure deficit, diurnal temperature fluctuations, heterogeneous soil conditions, and biotic factors that cannot be fully simulated in a controlled environment [26]. Studies have shown that plant physiological responses in controlled environment settings often differ from those observed in the field. For example, while trends such as altered photosynthesis or stomatal conductance may appear in both settings, their intensity and consistency can vary significantly [27,28,29]. This suggests that, despite the similarities, results from controlled environments should be cautiously interpreted when used to predict field performance [25,29]. The extent to which growth chamber and greenhouse data align with field outcomes has been explored only to a limited extent, leaving a gap between laboratory and field-based research [26]. Reflecting on differences between controlled and field research, Kohler (2002) [30] described this disconnect as a “glass wall” and a significant barrier in crop improvement.

While both controlled and field studies have contributed to our understanding of stress-related physiological responses of crops, these environments are often studied in isolation [11,12,21,24]. Growth chamber studies usually focus on early vegetative growth and apply stress in a reductionist form, which is acute, sudden, and for short durations [26,31,32]. In the field, stress usually develops gradually and can vary in intensity and duration. Plants often face more than one stress at once in real-field conditions, unlike the single stress condition commonly used in controlled studies [32]. As a result, the extent to which controlled-environment performance reflects field outcomes remains uncertain. To understand genotypic resilience, it’s essential to connect how plants respond to stress during early growth in controlled settings with how they perform during the critical reproductive stage in the field. Since stress effects can shift substantially as plants develop, bridging early-stage stress responses observed in controlled environments with later-stage field performance is essential for selecting genotypes with consistent and transferable tolerance traits. This study aimed to address this challenge by evaluating five commercial Virginia-type peanut cultivars under individual and combined heat and drought stress in controlled growth chamber conditions, followed by validation under combined stress in the field. The specific objectives were to (i) assess whether combined heat and drought stress imposes greater physiological impact than their individual stressors. (ii) Assess the extent to which physiological responses observed under growth chamber conditions are consistent with performance under field-relevant conditions. (iii) Identify better-performing commercial cultivars with greater tolerance to combined heat and drought stress across environments.

## 2. Materials and Methods

### 2.1. Plant Materials

Five high-oleic Virginia-type peanut cultivars, ‘Bailey II’, ‘Emery’, ‘NC 20’, ‘Sullivan’ (developed by North Carolina State University), and ‘Walton’ [33], were used in this study. These are the cultivars that are commercially grown in the Virginia-Carolina (VC) peanut belt. The agronomic traits and pedigree information for these cultivars are detailed in the Virginia Peanut Production Guide by Balota et al. [34]. These cultivars exhibit moderate resistance to Cylindrocladium black rot (*Cylindrocladium parasiticum*), early leaf spot (*Passalora arachidicola*) and late leaf spot (*Nothopassalora personata*), sclerotinia blight (*Sclerotinia minor*), and *Tomato spotted wilt virus* (genus Tospovirus; family Bunyaviridae) [34]. However, limited information is available regarding their tolerance to heat and drought stress, apart from Walton, which has been reported to perform better under extreme drought conditions [33].

### 2.2. Growth Chamber Experiment

**Figure 1:**
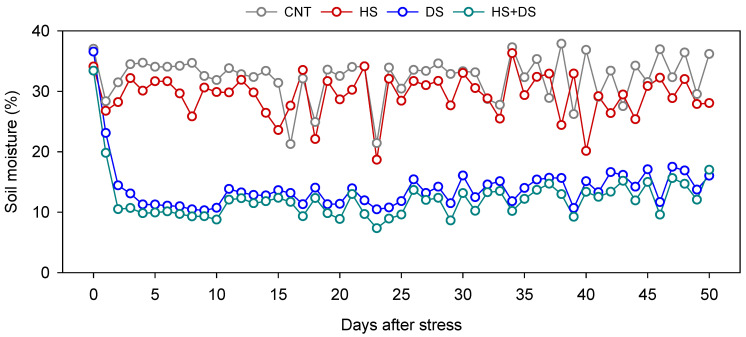
Percentage of soil moisture maintained in the growth chamber study, including five peanut cultivars and four treatments: CNT–control, HS–heat stress, DS–drought stress, HS+DS–combined heat and drought stress.

A controlled environment study was conducted during the spring of 2024 at the growth chamber facilities in Latham Hall, Virginia Tech, Blacksburg, VA, USA (37°13′ N, 80°25′ W). Seeds were sown in seedling trays filled with a 3:1 soil-to-sand mixture using Metromix soil medium (Sun Gro Horticulture, Agawam, MA, USA) and maintained under a 12 h photo period at 25 °C with a light intensity of 300 µmol m^−2^ s^−1^. Thirty days after planting (DAP), uniform sized seedlings were transplanted into 5-inch seed pots containing the same Metromix medium. The pots were then transferred to Conviron growth chambers (Conviron, Pembina, ND, USA) and arranged on foam blocks placed within trays to ensure proper drainage. The study utilized two growth chambers, one designated for the control (CNT) and drought stress (DS) treatments, and the other for heat stress (HS) and combined heat and drought stress (HS+DS) treatments. The day/night temperature regimes were maintained at 30/25 °C for CNT and DS and 40/35 °C for HS and HS+DS. The heat stress regime consisted of 40 °C during the day and 35 °C at night to simulate continuous heat stress conditions. The experiment followed a completely randomized design with four treatments, five peanut cultivars, and five replications per treatment, resulting in a total of 100 experimental units (Supplementary Figure S1). Soil moisture requirements for each treatment were determined using the gravimetric method [35]. Soil moisture levels were monitored daily using a digital soil moisture meter (Yinmik, Jinan, China). Pots in the CNT and HS treatments were gravimetrically maintained at approximately 30% volumetric water content (VWC, ~80% field capacity), while those in the DS and HS+DS treatments were maintained at ~15% VWC (~40% field capacity), and the stress period continued for 50 days (Figure 1).

### 2.3. Field Experiment

A field experiment was conducted during the summer of 2024 at the Tidewater Agricultural Research and Extension Center, Virginia Tech, located in Suffolk, VA, USA (36°39′ N, 76°44′ W). The soil at the site is classified as Eunola (fine-loamy, siliceous, semiactive, thermic Aquic Hapludults) [36]. Seeds were sown on 17 May 2024, in two-row plots 2.43 m long with 0.9 m row spacing and a seeding rate of 18 seeds m^−2^. Prior to planting, the land was tilled, and raised beds of 15 cm height were prepared. The trial was arranged in a randomized complete block design (RCBD) with four replications per treatment. A total of 40 experimental units were established, consisting of two treatments across five peanut cultivars with four replications each (2 treatments × 5 cultivars × 4 replications). Plants were initially grown under rainfed conditions until 2 July 2024 (45 DAP and beginning flowering—R1 growth stage). Due to lack of rainfall, a supplemental irrigation of 25 mm was applied four weeks after planting to support biomass accumulation before stress initiation. This ensured better canopy cover, minimizing water loss through evaporation from the soil, allowing transpiration as the main source of soil water depletion. At 45 DAP, rainout shelters (RS) were installed over 50% of the plots to impose the HS+DS treatment. The remaining plots were left exposed to natural conditions and served as rainfed/CNT treatment. The shelters were constructed using translucent clear plastic sheeting tarp mounted on a metal frame, allowing sunlight penetration while preventing rainfall. The shelters reduced airflow and increased daytime temperatures compared with open rainfed conditions, thereby enhancing the heat stress in addition to inducing drought stress by excluding precipitation. Weather conditions in the open rainfed (CNT) plots were monitored using an ATMOS 41 weather station (METER Group, Pullman, WA, USA), which recorded air temperature (°C). In the HS+DS plots, microclimate data were recorded using HOBO data loggers (LI-COR Biosciences, Lincoln, NE, USA) to capture air temperature and humidity under shelter conditions. Soil moisture (m^3^ m^−3^ VWC) and soil temperature (°C) were measured using TEROS 12 sensors (METER Group, Pullman, WA, USA) installed at a depth of 20 cm in the root zone. Data were logged continuously using ZL6 Pro data loggers (METER Group, Pullman, WA, USA). The stress treatment was maintained for 10 weeks (70 days) and concluded on 9 September 2024, allowing the plants to recover before harvest (Supplementary Figure S2).

### 2.4. Data Collection

Stomatal conductance (g_sw_, mol m^−2^ s^−1^), transpiration (E, mmol m^−2^ s^−1^), and leaf temperature difference (leaf temperature minus air temperature, T_diff_, °C) were measured using the LI-600 porometer (LI-COR Biosciences, Lincoln, NE, USA). Data were obtained using the manual setting on the LI-600. Leaves were clamped for approximately 30 s until g_sw_ values stabilized, after which values were recorded.

In the growth chamber experiment, measurements were taken from the third fully expanded leaf from the apex of each plant. Data was collected every third day for 35 days following stress initiation, spanning from the vegetative to early reproductive stages (approximately V5 to R1). While nighttime temperatures were elevated, all measurements were performed between 10:00 and 13:00 h focusing on daytime physiological responses.

In the field experiment, measurements were conducted weekly over 8 weeks (R1 to R5) following stress initiation, covering the reproductive growth stages. Similarly to the growth chamber, measurements were taken between 10:00 and 13:00 h. In each two-row plot, two representative plants were sampled, and the average of the two readings was used in the analysis.

For comparison analysis between growth chamber and field conditions, the first eight measurement time points from the CNT and HS+DS treatments in the growth chamber were aligned with eight weekly sampling dates following stress initiation in the field experiment under the same treatments. Due to space limitations in the growth chamber, measurements focused on the vegetative to early reproductive stages, while the field experiment prioritized reproductive growth stages. Trait relationships within and across treatments in the growth chamber and field experiments were assessed using Pearson correlation and principal component analysis (PCA) to evaluate stability in physiological responses across environments.

### 2.5. Statistical Analysis

Data analyses were conducted using R (version 4.4.3; R Core Team, 2025) implemented in RStudio (version 2024.09.0). A linear mixed-effects model was fit using the ‘lmer()’ function from the ‘lme4’ package, with days or weeks after stress, treatment, cultivar, and their interactions as fixed effects, and replication as a random effect and analysis of variance (ANOVA) for these fixed effects was conducted using the ‘anova()’ function from the ‘lmerTest’ package. Tukey’s Honest Significant Difference (HSD) post hoc comparisons were conducted at a significance level of 0.05 using the ‘emmeans’ and ‘multcomp’ packages [37,38,39,40]. Graphical representations were created using the SigmaPlot software (version 14.5; Systat Software, San Jose, CA, USA). To compare responses between the field and growth chamber experiments, a subset of data from the growth chamber was used. This subset included the first eight sampling time points under the CNT and HS+DS treatments, which were aligned with corresponding sampling dates in the field (as described in Section 2.4). These were compared using Pearson correlations and PCA. Pearson correlation coefficients among traits were calculated and visualized using the ‘corrplot’ package [41]. PCA was conducted using the ‘FactoMineR’ package [42] and visualized with ‘factoextra’ [43].

## 3. Results

### 3.1. Microclimatic Conditions During the Experiments

**Figure 2:**
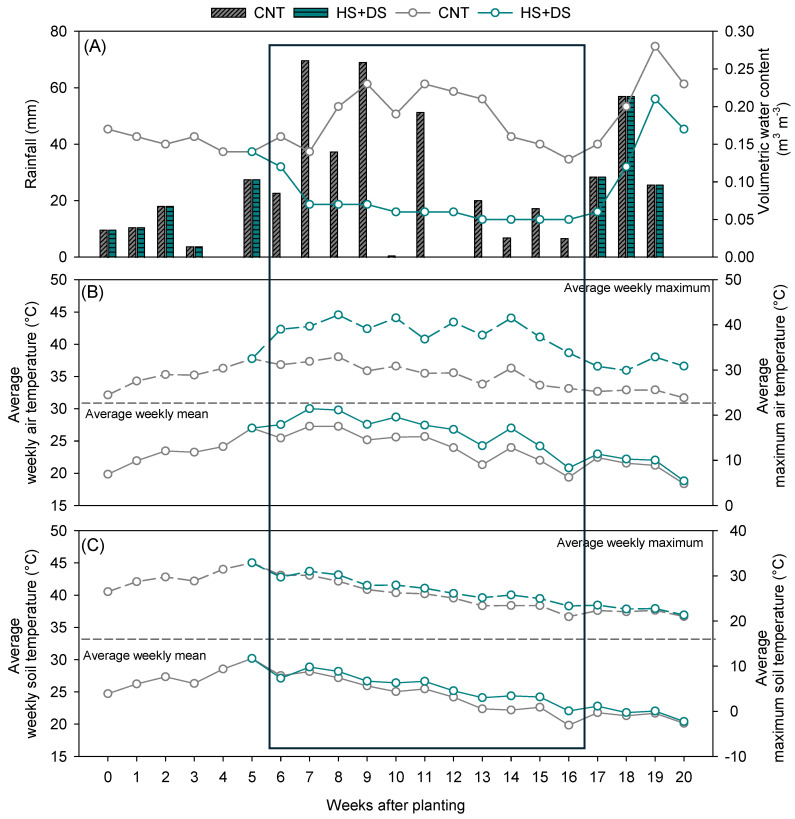
(**A**) Amount of rainfall (**left** axis) and volumetric water content (**right** axis) in the field study from peanut planting to 20 weeks after planting. (**B**) Weekly average and maximum air temperature. (**C**) Weekly average and maximum soil temperature during the growing season of peanuts at the field site, Tidewater Agricultural Research and Extension Center, Suffolk, VA, USA. The highlighted region from 6 to 16 weeks after planting indicates the stress-imposition period.

In the growth chamber study, soil moisture content maintained after stress imposition for all four treatments is shown in Figure 1. In the field experiment, variation in natural weather conditions influenced the degree of stress experienced across treatments (Supplementary Table S1). Over the crop growth period, CNT plots received a total of 485 mm of rainfall, while HS+DS plots received substantially less rainfall, totaling only 182 mm (Figure 2A). During the 10-week stress period, the average VWC in CNT plots was 0.18 m^3^ m^−3^, whereas HS+DS plots maintained much lower values, averaging 0.06 m^3^ m^−3^ (Figure 2A). Average daily air temperatures were 24 °C in CNT and 27 °C in HS+DS plots (Figure 2B). Maximum air temperatures reached 30 °C in CNT and up to 39 °C in HS+DS plots (Figure 2B). Soil temperature followed a similar trend, with average values of 25 °C in CNT and 26 °C in HS+DS, while maximum soil temperatures were slightly higher in HS+DS (27 °C) compared to CNT (26 °C) (Figure 2C).

### 3.2. Leaf Responses to Heat and Drought Stress Under Controlled Environment

The Linear mixed effects model ANOVA results showed that days after stress (DAS) significantly affected g_sw_ (F_(10,880)_ = 24.8, *p <* 0.001), E (F_(10,880)_ = 28.1, *p <* 0.001), and T_diff_ (F_(10,880)_ = 22.8, *p <* 0.001). Similarly treatment (T) significantly influenced g_sw_ (F_(3,880)_ = 84.3, *p <* 0.001), E (F_(3,880)_ = 89.1, *p <* 0.001), and T_diff_ (F_(3,880)_ = 61.4, *p <* 0.001), and the interaction between DAS and T was significant for g_sw_ (F_(30,880)_ = 4.5, *p <* 0.001), E (F_(30,880)_ = 4.1, *p <* 0.001), and T_diff_ (F_(30,880)_ = 12.5, *p <* 0.001). Cultivar (C) had a significant effect on g_sw_ (F_(4,880)_ = 5.3, *p <* 0.001) and E (F_(4,880)_ = 5.6, *p <* 0.001) (Table 1).

**Figure 3:**
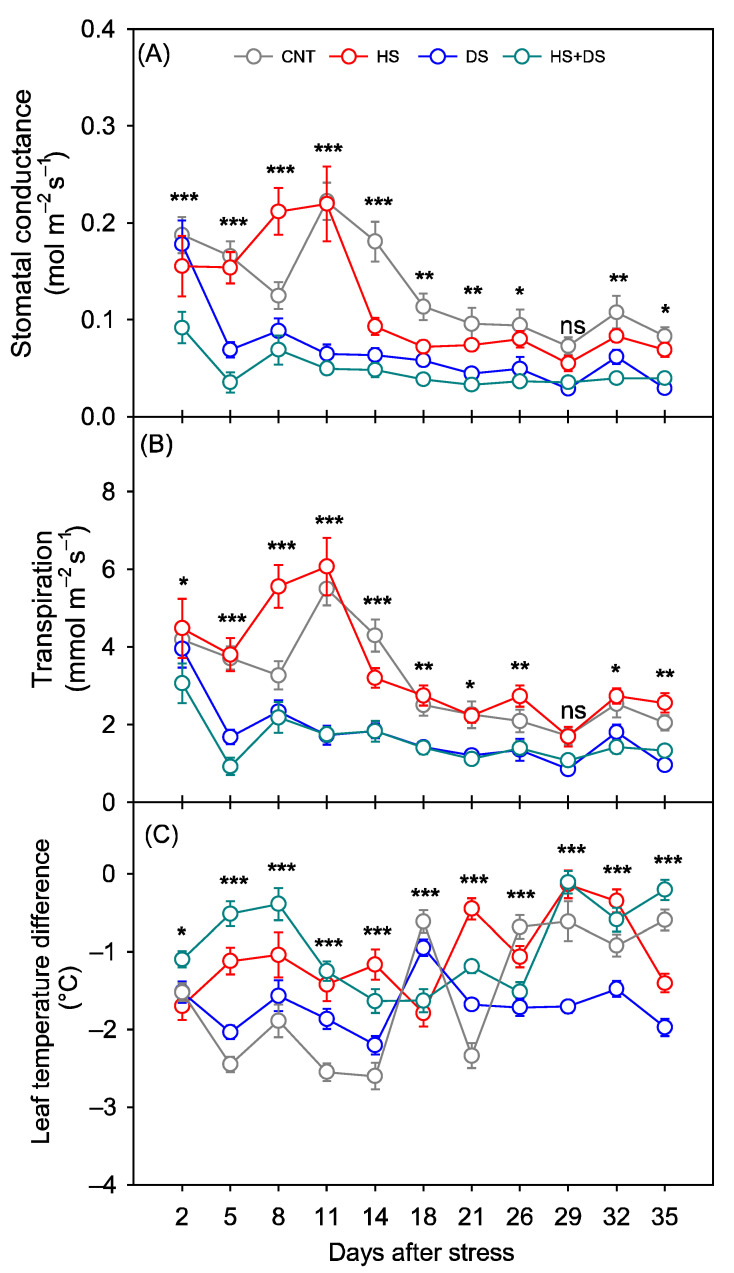
Effects of heat and drought stress on (**A**) stomatal conductance, (**B**) transpiration, and (**C**) leaf temperature difference in peanuts over time in the growth chamber study. *, **, and *** indicate significant difference at *p* < 0.05, *p* < 0.01, and *p* < 0.001, respectively, and ns indicates non-significance among treatments each day after stress initiation. Each data point represents mean ± SE of twenty-five replicates. CNT-control, HS-heat stress, DS-drought stress, HS+DS-combined heat and drought stress.

**Figure 4:**
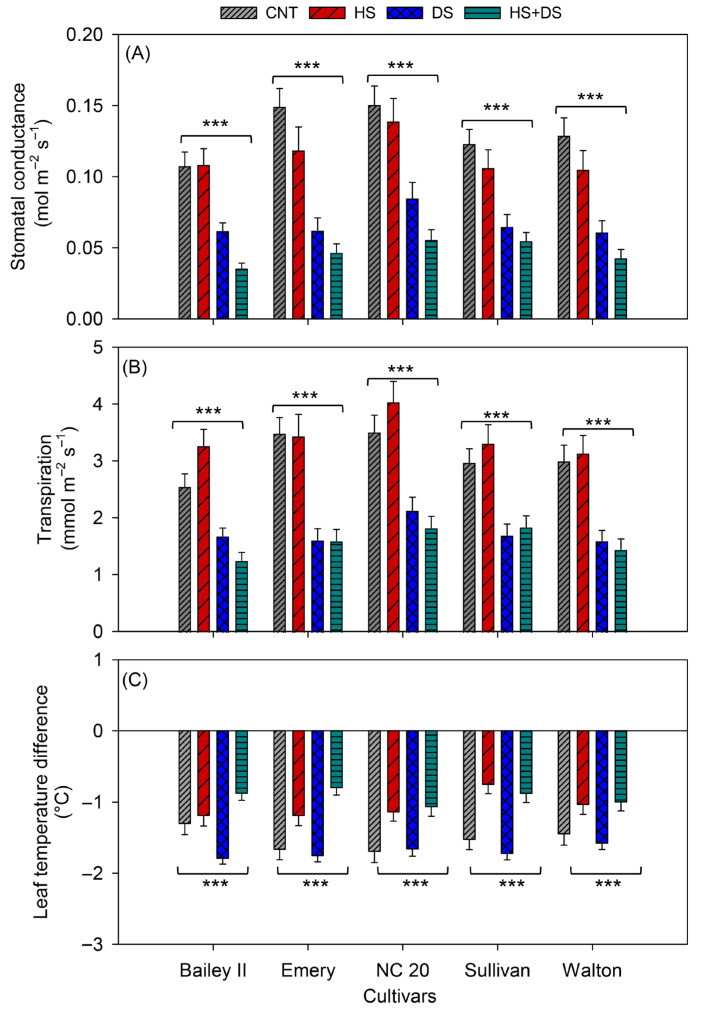
Effects of heat and drought stress on (**A**) stomatal conductance, (**B**) transpiration, and (**C**) leaf temperature difference in peanut cultivars in the growth chamber study. *** indicates significant difference at *p* < 0.001, respectively, and ns indicates non-significance among treatments within each cultivar. Each vertical bar represents mean ± SE of 55 observations, obtained from measurements on five replicates across 11 time points. CNT-control, HS-heat stress, DS-drought stress, HS+DS-combined heat and drought stress.

Across treatments, cultivars showed clear differences in g_sw_ and E, while differences in T_diff_ were non-significant and mostly influenced by the treatment itself (Table 1). Under combined HS+DS, all cultivars exhibited lower g_sw_, E, and higher T_diff_ compared to the other treatments from 2–35 DAS (Figure 3A–C). On average of 35 DAS, NC 20 maintained the highest g_sw_ (0.06 mol m^−2^ s^−1^) and E (1.8 mmol m^−2^ s^−1^), along with the most negative T_diff_ −1.06 °C, consistent with better physiological performance and evaporative cooling under stress (Figure 4A–C). Sullivan followed closely in gas exchange (g_sw_: 0.06 mol m^−2^ s^−1^; E: 1.8 mmol m^−2^ s^−1^) but had a warmer canopy than NC 20 (T_diff_: −0.9 °C) (Figure 4A–C). Emery and Walton showed intermediate responses, while Bailey II had the lowest g_sw_ (0.04 mol m^−2^ s^−1^) and E (1.3 mmol m^−2^ s^−1^), and limited cooling capacity (T_diff_: −0.9 °C), consistent with increased susceptibility to combined stressors (Figure 4A–C).

Stomatal conductance and E values were generally higher under the DS treatment than for those measured under the HS+DS treatment, but below the CNT and HS treatments (Figure 3A,B). Under DS, NC 20 ranked highest (g_sw_: 0.09 mol mm^−2^ s^−1^; E: 2.1 mmol m^−2^ s^−1^), with Sullivan, Walton, Bailey II, and Emery following in descending order. T_diff_ values ranged from −1.6 °C (Walton) to −1.8 °C (Bailey II), with no clear cultivar-specific pattern (Figure 4A,B). These results suggest that while most cultivars experienced reduced gas exchange under drought, canopy cooling remained relatively effective.

All cultivars showed elevated g_sw_ values under the HS treatment when compared with DS and HS+DS, but not relative to the CNT treatment. NC 20 showed the highest values (g_sw_: 0.1 mol m^−2^ s^−1^; E: 4.0 mmol m^−2^ s^−1^), followed by Emery and Bailey II (Figure 4A,B). T_diff_ values under HS ranged from −1.1 °C (NC 20) to −0.8 °C (Sullivan), indicating that while g_sw_ increased, cooling was less consistent across cultivars (Figure 4C). In particular, cv. Sullivan exhibited an elevated g_sw_ value and a warmer canopy, suggesting that water loss and cooling may be decoupled in this cultivar.

In summary, NC 20 consistently outperformed other cultivars across all stress conditions, maintaining high gas exchange and effective canopy cooling. Sullivan showed strong leaf gas exchange but less effective cooling under heat compared to NC 20 (Figure 4). Emery exhibited robust performance under HS and CNT but was significantly affected by DS. Walton maintained moderate performance across all treatments, while Bailey II was the most susceptible cultivar, especially under combined stressors. These results highlight NC 20 and Sullivan as the most resilient cultivars to heat, drought, and the combination thereof, under controlled conditions.

### 3.3. Leaf Responses Under Field Conditions to Heat and Drought Stress

In the field study, linear mixed effect model ANOVA showed that weeks after stress had a strong influence on g_sw_ (F_(7,520)_ = 51.6, *p <* 0.001), E (F_(7,520)_ = 75.4, *p <* 0.001), and T_diff_ (F_(7,520)_ = 21.8, *p <* 0.001). Treatment significantly affected g_sw_ (F_(1,520)_ = 39.1, *p <* 0.001) and E (F_(1,520)_ = 79.2, *p <* 0.001), but not T_diff_ (F_(1,520)_ = 0.1, *p >* 0.05). Cultivar had a significant effect on g_sw_ (F_(4,520)_ = 4.1, *p <* 0.01), E (F_(4,520)_ = 2.4, *p <* 0.05) and T_diff_ (F_(4,520)_ = 0.01, *p <* 0.05). The interaction between WAS and T was significant for all three traits g_sw_(F_(7,520)_ = 13.7, *p <* 0.001), E (F_(7,520)_ = 8.9, *p <* 0.001), and T_diff_ (F_(7,520)_ = 4.9, *p <* 0.001), indicating that treatment effects changed over time. The WAS × C interaction was significant only for g_sw_ (F_(28,520)_ = 2.7, *p <* 0.001), suggesting cultivar differences in stomatal regulation over time (Table 2).

**Figure 5:**
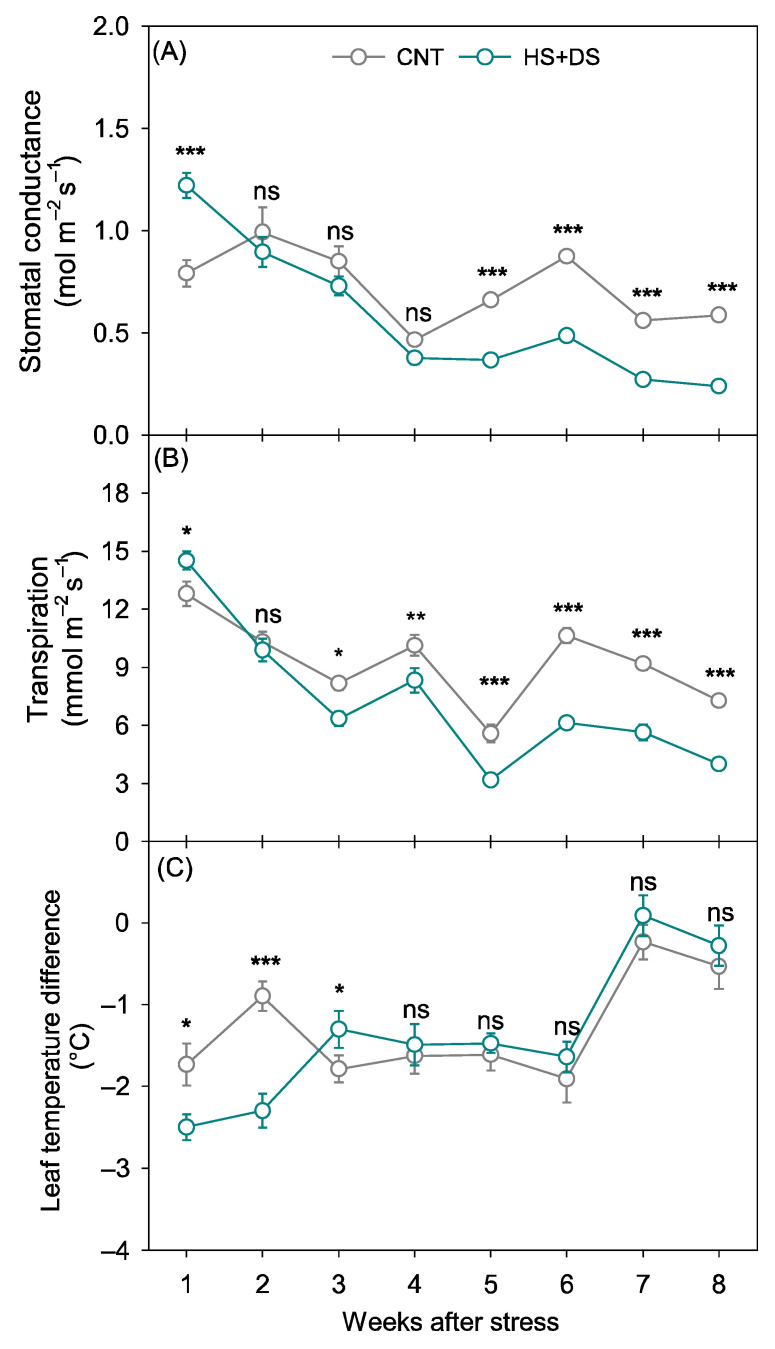
Effects of heat and drought stress on (**A**) stomatal conductance, (**B**) transpiration, and (**C**) leaf temperature difference in peanuts over time in the field study when stress was imposed by rain exclusion shelters. *, **, and *** indicate significant difference at *p* < 0.05, *p* < 0.01, and *p* < 0.001, respectively, and ns indicates non-significance, among treatments within each week after stress initiation. Each data point represents mean ± SE of forty replicates. CNT-control, HS+DS-combined heat and drought stress.

Combined heat and drought stress caused substantial reductions in both g_sw_ and E compared to the CNT. The average g_sw_ in CNT plots was 0.7 mol m^−2^ s^−1^, while under HS+DS it declined to 0.55 mol m^−2^ s^−1^, representing a 21% reduction (Figure 5A). This decline was most prominent between weeks 1 and 5 after stress imposition. During weeks 1 and 2, g_sw_ values under HS+DS were higher than those in the open field, averaging 1.2 and 0.9 mol m^−2^ s^−1^, respectively, for the HS+DS, while it was 0.8 and 1.0 mol m^−2^ s^−1^ for CNT (Figure 5A). However, by week 5, g_sw_ under HS+DS had been reduced to half of that in CNT and remained consistently low through weeks 5 to 8 (Figure 5A).

Similarly to growth chamber study, E mirrored the trends observed in g_sw_. The average E in CNT was 9.2 mmol m^−2^ s^−1^, while HS+DS reduced E by 24% to 7.0 mmol m^−2^ s^−1^. During week 1, E under HS+DS reached 14.5 mmol m^−2^ s^−1^ compared to 12.8 mmol m^−2^ s^−1^ in the CNT, a 13% increase (Figure 5B). By week 4, E under HS+DS had declined to 6.8 mmol m^−2^ s^−1^, a 26% reduction compared to the CNT, and this lower rate persisted through the end of the study (Figure 5B).

In average of 8 weeks of stress, T_diff_ was slightly more negative under HS+DS (−1.3 °C) compared to CNT (−1.2 °C), indicating marginally cooler leaves under stress, although this difference was not statistically significant. The lower T_diff_ under HS+DS during weeks 1 and 2 was associated with higher transpiration, suggesting effective evaporative cooling early after stress imposition (Figure 5C). However, from weeks 3 to 8, T_diff_ values under HS+DS became less negative, indicating relatively warmer leaves than in CNT, reflecting a reduction in cooling capacity as g_sw_ and E declined with ongoing stress (Figure 5A–C).

**Figure 6:**
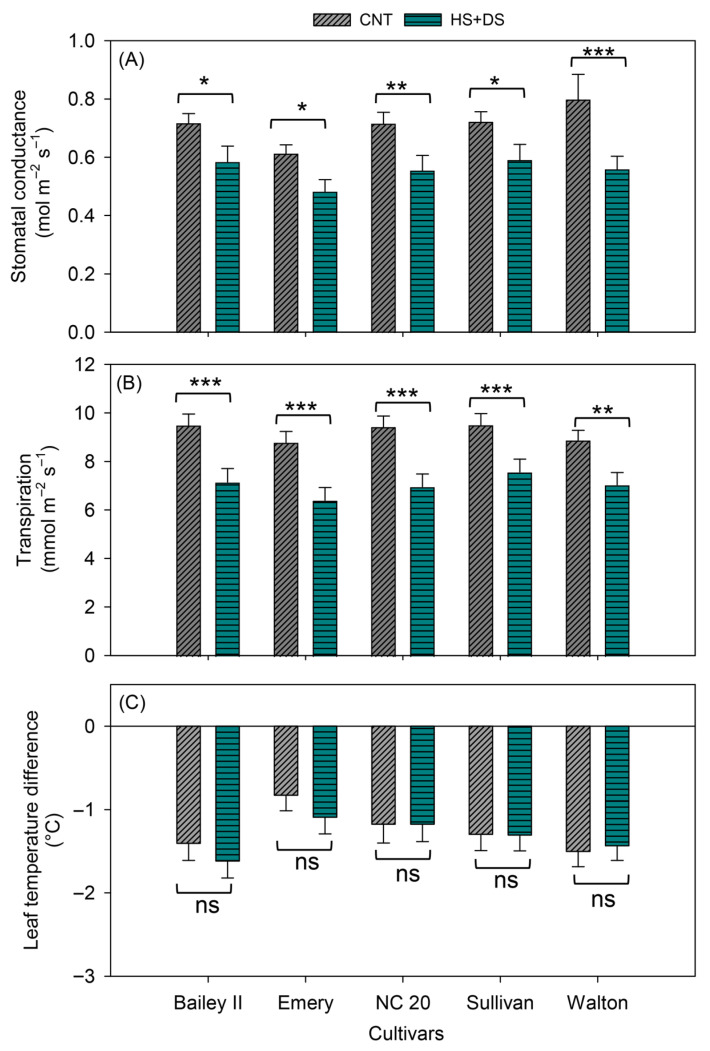
Effects of heat and drought stress on (**A**) stomatal conductance, (**B**) transpiration, and (**C**) leaf temperature difference in peanut cultivars in the field study when stress was imposed by rain exclusion shelters. *, **, and *** indicate significant difference at *p* < 0.05, *p* < 0.01, and *p* < 0.001, respectively, and ns indicates non-significance among treatments within each cultivar. Each vertical bar represents mean ± SE of 64 observations, obtained from measurements on eight replicates across 8 time points. CNT-control, HS+DS-combined heat and drought stress.

Among cultivars, Sullivan showed a better overall performance in the field, maintaining high g_sw_ (0.6 mol m^−2^ s^−1^), E (7.6 mmol m^−2^ s^−1^), and stable canopy cooling (T_diff_ −1.3 °C) (Figure 6A–C). Bailey II also performed well, with moderate reductions in gas exchange rate but the most negative T_diff_ (−1.6 °C) under prolonged stress, indicating a strong cooling capacity under this environment (Figure 6A-C). NC 20 showed moderate resilience, with steady T_diff_ (−1.2 °C) but reduced overall gas exchange rates (Figure 6A–C). Walton retained relatively high cooling capacity (−1.4 °C T_diff_) despite a moderate decline in g_sw_ and E (Figure 6A-C). In contrast, Emery exhibited the lowest g_sw_ (0.5 mol m^−2^ s^−1^) and E (6.4 mmol m^−2^ s^−1^), and the least negative T_diff_ (−1.1 °C), indicating poor performance under stress in the field (Figure 6A–C). Overall, Sullivan and Bailey II were the most resilient cultivars in the field under combined stress, while Emery was the most susceptible.

### 3.4. Comparative Analysis

To compare responses across environments, we analyzed the average g_sw_, E, and T_diff_ in both the growth chamber and field. Despite differences in developmental stage and environmental conditions, consistent reductions in g_sw_ and E under HS+DS were observed in both settings, with a greater magnitude of decline in the growth chamber. Pearson correlation and PCA revealed that genotypes under stress conditions showed partial consistency across environments, suggesting that these physiological traits may be predictive of stress responses in both vegetative and reproductive stages.

**Figure 7:**
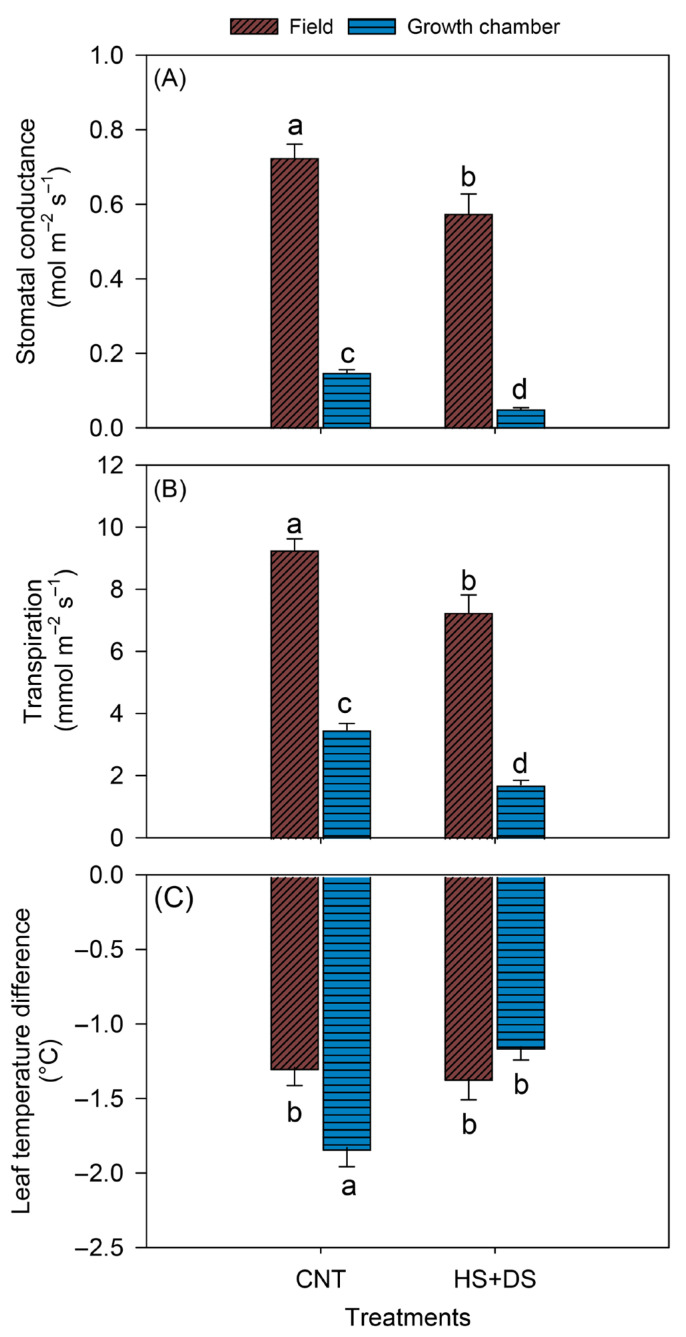
A comparison between field and growth chamber peanut stress responses on (**A**) stomatal conductance, (**B**) transpiration, and (**C**) leaf temperature difference. Data from five cultivars and up to 26 days after stress from growth chamber and 8 weeks after stress from field were combined for this analysis. The vertical bars represent mean ± SE. Different letters indicate significant differences between treatments across days after stress at *p* < 0.05 by Tukey’s honestly significant difference test. Treatments were CNT-control, HS+DS-combined heat and drought stress.

Under CNT, g_sw_ was higher by 79% in the field (0.7 mol m^−2^ s^−1^) than in the growth chamber (0.15 mol m^−2^ s^−1^) (Figure 7A). Similarly, HS+DS treatment showed 91% lower values of g_sw_ in the growth chamber (0.05 mol m^−2^ s^−1^) compared to the field (0.55 mol m^−2^ s^−1^) (Figure 7A). Transpiration rate was 9.2 mmol m^−2^ s^−1^ in the field and 3.5 mmol m^−2^ s^−1^ in the growth chamber, a 63% difference in range under CNT (Figure 7B), while for the HS+DS the variation was 76% (7.2 mmol m^−2^ s^−1^ in the field and 1.7 mmol m^−2^ s^−1^ in the growth chamber) (Figure 7B). Unlike g_sw_ and E, T_diff_ had comparable values between field and growth chamber. After the initial 8 sampling times, the growth chamber plants had 47% relatively cooler leaves (−1.8 °C) than the field plants (−1.2 °C) under CNT (Figure 7C). Conversely, under HS+DS conditions, the growth chamber plants exhibited 13% increased T_diff_ (cooler leaves) compared to the field-grown plants (Figure 7C).

**Figure 8:**
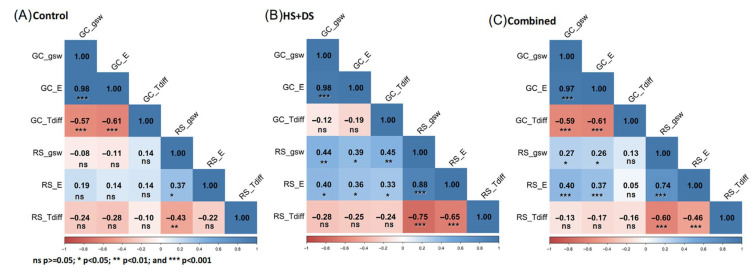
Pearson correlation matrices of physiological traits measured in growth chamber (GC) and rainout shelter (RS) experiments under three conditions: (**A**) control, (**B**) combined heat and drought stress (HS+DS), and (**C**) a combination of both control (panel (**A**)) and HS+DS (panel (**B**)). Blue indicates positive correlations and red indicates negative correlations; the color intensity reflects the strength of the relationship. Correlation coefficients close to ±1 represent strong relationships, while values near 0 indicate weak or no correlation. Statistical significance is denoted as follows: * *p* < 0.05, ** *p* < 0.01, *** *p* < 0.001; ns = not significant.

Under CNT conditions, g_sw_ and E measurements from the field and growth chamber showed no significant correlation (Figure 8A), indicating limited alignment between environments under well-watered conditions, likely due to greater variability in the field. Under HS+DS, significant positive correlations were observed between growth chamber and field for g_sw_ (r = 0.44, *p <* 0.01) and E (r = 0.36, *p <* 0.05), while T_diff_ remained uncorrelated between the two environments (Figure 8B). When data from both treatments were combined, significant but moderate correlations were observed for g_sw_ (r = 0.27, *p <* 0.05) and E (r = 0.37, *p <* 0.001) between the growth chamber and field, whereas T_diff_ showed no significant relationship between environments (Figure 8C).

**Figure 9:**
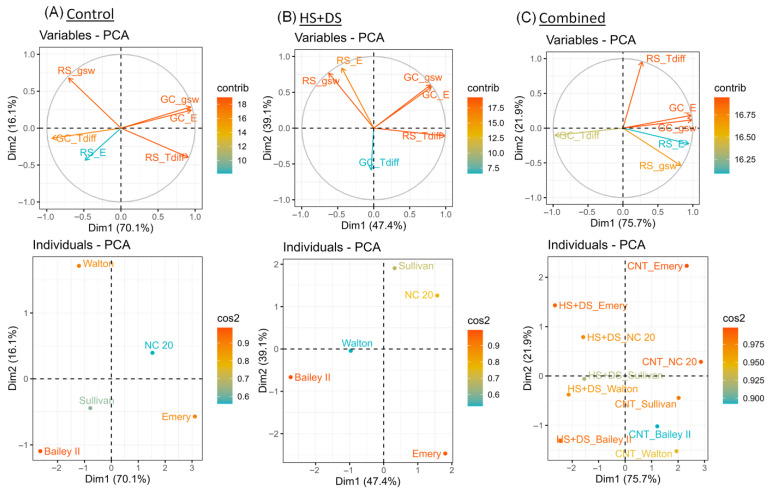
Principal component analysis (PCA) of five peanut cultivars under different environmental conditions. The top row shows variable factor maps on the first two principal components (Dim.1 and Dim.2). The bottom row shows corresponding cultivar score plots under: (**A**) control conditions, (**B**) combined heat and drought stress (HS+DS), and (**C**) all conditions combined (control (panel **A**), heat, and drought stress (panel **B**)). Data were obtained from growth chamber (GC) and rainout shelter (RS) field experiments. Color gradients and vector lengths represent variable contributions to the principal components.

Principal component analysis results supported and expanded the correlation analysis by providing a multivariate view of relationships among gas exchange and thermal traits. Under CNT conditions, the first principal component (PC1) explained 70.1% of the variance (Figure 9A). Genotypic distribution along the PC1 showed distinct patterns: Emery was aligned with the field T_diff_, suggesting higher leaf temperature and lower gas exchange rates, while NC 20 was closely associated with g_sw_ and E in the growth chamber (Figure 9A). Under the HS+DS treatment, PC1 and PC2 explained 86.5% of the variance (Figure 9B). Gas exchange traits from both environments loaded positively on PC1. Field T_diff_ also loaded positively on PC1, while growth chamber T_diff_ loaded negatively on PC2, reflecting environment-specific thermal responses. Bailey II grouped with the field gas exchange traits but was separated from growth chamber traits, highlighting its contrasting performance across environments (Figure 9B). Emery was positioned opposite the gas exchange traits, indicating high susceptibility to stress. NC 20 and Sullivan remained strongly associated with g_sw_ and E, reflecting consistent stress tolerance across environments. In the combined PCA across treatments, PC1 explained 75.7% of the variance, driven by gas exchange traits, while field T_diff_ contributed primarily to PC2 (Figure 9C). Cultivars were separated by treatment along PC1, control samples showing positive scores, and HS+DS samples showing negative scores. Cultivar-level comparisons revealed consistent patterns as NC 20 and Sullivan maintained stable positions across treatments, consistent with resilience to these stressors.

## 4. Discussion

Environmental stressors such as heat and drought are major limiting factors to crop productivity, particularly in semi-arid regions [44]. The physiological adaptability of crops, including peanuts, is important for maintaining yield stability under these challenging conditions [45]. In this study, we compared the physiological responses of five Virginia-type peanut cultivars to heat and drought stress in a controlled (growth chamber) environment during vegetative growth stages and a semi-controlled (rainout shelter) field environment during reproductive stages. While the stress conditions were not directly parallel with timing or intensity, we used comparable physiological measurements (g_sw_, E, and T_diff_) across both environments to examine genotypic responses. This comparison allowed us to explore whether physiological patterns observed under controlled, early-stage conditions reflect broader stress resilience tendencies under more variable, field conditions. Although not a direct comparison, this approach contributes to bridging controlled-environment screening with field performance assessment, an important step toward identifying genotypes with stress tolerance across developmental stages and environments.

Our results revealed that the severity of stress impacts followed the order of HS < DS < HS+DS in the growth chamber, with HS causing only minor reductions in g_sw_ and even an increase in E (Figure 3A,B). DS alone led to more pronounced g_sw_ and E declines than HS, while the combined HS+DS treatment resulted in relatively greater reductions than the individual treatments (Figure 3 and Figure 4). Notably, from 26 to 35 DAS, plants under DS exhibited more negative T_diff_ and lower E (Figure 3B,C). Most studies indicate that reduced E under DS typically results in warmer canopies and less negative T_diff_ values, since evaporative cooling is diminished [46,47]. While morphological adaptations such as leaf rolling or orientation can provide some passive cooling [48,49], these effects are generally modest compared to the cooling achieved through transpiration. Therefore, our observation of cooler leaves despite low transpiration rate is atypical, suggesting that our unique experimental conditions or additional, less-documented adaptive responses may be involved.

In the field, where only rainfed CNT and HS+DS treatments were compared, similar but less severe declines were observed under stress (in both g_sw_, E) (Figure 5A,B). Lower g_sw_ and E consistently led to higher T_diff_, in both environments (growth chamber and field), indicating that reduced stomatal opening limited transpirational cooling, driving leaf temperature increases [50]. This relationship aligns with the fundamental role of stomata in regulating water loss and leaf temperature under heat and drought stress [51]. Although limited data are available on the interactive effects of heat and drought stress in peanut (Hamidou et al., 2013) [1], the patterns observed in this study align with findings in other major crops, such as wheat, soybean, maize, and cotton (*Gossypium hirsutum* L.) [16,17,18,52].

Both environments showed overall reductions in g_sw_, and E under HS+DS; however, the magnitude of decline was greater in the growth chamber than in the field. This difference may be due to resource limitations in a controlled environment, e.g., restricted root growth and reduced water uptake. Additionally, the absence of a developed canopy in the growth chamber likely contributed to increased radiation load, as field-grown plants benefited from self-shading and moderated microclimates that can reduce thermal stress [32,53]. It is also important to note that the magnitude of heat stress differed between environments. While the growth chamber maintained constant high temperatures (e.g., 40 °C/35 °C day/night), field temperatures fluctuated and were often lower, potentially resulting in less cumulative thermal stress. The field-grown plants experienced a prolonged dry period of about a week at four weeks after planting and right before the coverage with the rain shelters. This early dry spell may have triggered a primed acclimation process in the field-grown plants, enhancing their tolerance to subsequent stress when covered by the rain shelters [54,55]. Such acclimation involves changes in gene expression and physiological function following repeated stress events, allowing plants to better cope with future stresses [54]. Early stress exposure has been associated with improved root development, water use efficiency, and photosynthetic stability in peanuts and other crops [54,56,57,58].

Plants in the growth chamber showed immediate declines in g_sw_ and E upon stress exposure, likely due to rapid water depletion and limited buffering capacity in the controlled setting [26,59]. Field-grown plants, which were more developmentally advanced than growth chamber plants at the onset of stress, initially maintained higher physiological activity, potentially due to greater soil moisture in the root zone at the initiation of stress and moderated microclimate from canopy cover [54,60]. As stress persisted, physiological activity declined below CNT treatment levels, indicating that prolonged exposure eventually diminished the effectiveness of initial compensatory responses. These contrasting patterns highlight the influence of environmental complexity and resource availability on the timing and magnitude of stress responses [32,54,56,60].

Cultivar-specific responses across growth environments revealed strong genotype-by-environment (G × E) interactions, emphasizing the importance of genetic background in peanut stress resilience. Under HS+DS in the growth chamber, NC 20 maintained the highest g_sw_ and E rates and achieved effective canopy cooling, indicative of active stomatal regulation and increased water-use efficiency relative to the other tested cultivars. These traits persisted under field stress, where NC 20 showed moderate reductions in gas exchange but retained thermal stability, suggesting constitutive stress tolerance mechanisms with low G × E variability, a pattern consistent with the stable plasticity model [61]. Sullivan also demonstrated strong gas exchange across environments, ranking second to NC 20 under controlled conditions, but in the field it outperformed NC 20 in both g_sw_ and E while maintaining favorable T_diff_. This suggests genotypes focusing on growth environment-specific adaptive plasticity and dynamic modulation of stress responses [62]. Although Bailey II, susceptible under controlled HS+DS (lowest g_sw_ and E), exhibited improved canopy cooling under field stress, indicating that its performance is more growth environment cued, a case of environment-induced plasticity. Emery showed robust gas exchange traits under heat alone but was among the weakest under drought and HS+DS across both environments, showing a high degree of stress susceptibility and a poor G × E profile. Walton maintained moderate physiological performance under all treatments but lacked standout resilience. Comparative analysis reinforced these findings, NC 20 and Sullivan were strongly associated with high g_sw_ and E in PCA, while Bailey II and Emery grouped with traits indicating lower gas exchange and canopy cooling, particularly under growth chamber stress (Figure 9). Correlation and PCA analyses showed that gas exchange and cooling were more closely linked in the field than in the growth chamber, suggesting that complex field conditions could alter the connection between traits and differences among cultivars [26]. Although these correlations show trait associations that hold true in different environments, they do not always mean that absolute trait values from growth chambers translate directly to field settings.

These insights align with the framework of functional phenotyping, which emphasize evaluating plant physiological traits under defined environmental conditions to inform crop selection and breeding for stress resilience [63,64]. By comparing cultivar performance across controlled and field environments, this study highlights how physiological traits g_sw_, E, and T_diff_ can reveal adaptive advantages critical for selecting cultivars suited to semi-arid conditions. Although no statistically significant cultivar-by-treatment interaction was observed, distinct cultivar-specific trends emerged across environments. Furthermore, stronger trait correlations under stress, compared to control treatments, support the hypothesis that plants exhibit similar survival-driven strategies under abiotic pressure [65]. While molecular tools dominate stress biology, this study reinforces the value of whole-plant physiological assessments to enable more effective and field-relevant crop improvement strategies for stress-prone environments.

## 5. Conclusions

This study compared the physiological responses of five Virginia-type peanut cultivars to heat and drought stress under controlled growth chamber and field conditions. The combined HS+DS treatment had a more negative effect on physiological traits than either stress alone in the growth chamber study. While both environments showed a declining trend in g_sw_ and E, the reduction was more pronounced under growth chamber environment. However, long-term growth chamber studies may be suboptimal for peanuts due to the plant’s extended growth cycle, expansive root and canopy development, and the inherent spatial and resource limitations of such systems. No single cultivar consistently outperformed others across both environments, but overall, NC 20 and Sullivan displayed a balance of stability and plasticity in trait expression across environments, positioning them as better cultivars for breeding programs targeting climate resilience. These findings support the existing hypothesis that cultivar performance under combined heat and drought stress is influenced by environment, growth stage, stress duration, and intensity. Incorporating yield-related traits in future analyses will provide a more comprehensive understanding of how stress affects overall productivity and quality.

## Figures and Tables

**Table 1:** F-statistics from a linear mixed effects model analysis of variance of the physiological traits, including stomatal conductance, transpiration, and leaf temperature difference, measured in the growth chamber study from five peanut genotypes across four treatments.

Traits		Stomatal Conductance					Transpiration					Leaf Temperature Difference				
SOV	Df	Sum Sq	Mean Sq	F Value	Pr (>F)		Sum Sq	Mean Sq	F Value	Pr (>F)		Sum Sq	Mean Sq	F Value	Pr (>F)	
DAS	10	1.3	0.1	24.8	<2.2 × 10^−16^	***	773.4	77.3	28.1	<2.2 × 10^−16^	***	140.4	14.0	22.8	<2.2 × 10^−16^	***
T	3	1.3	0.4	84.3	<2.2 × 10^−16^	***	727.3	242.3	89.1	<2.2 × 10^−16^	***	113.4	37.8	61.4	<2.2 × 10^−16^	***
C	4	0.1	0.03	5.3	0.0003	***	61.1	15.3	5.6	0.0002	***	4.0	1.0	1.6	0.2	ns
DAS × T	30	0.7	0.02	4.5	3.4 × 10^−14^	***	341.2	11.4	4.1	<1.5 × 10^−12^	***	230.6	7.7	12.5	<2.2 × 10^−16^	***
DAS × C	40	0.1	0.003	0.6	1.0	ns	65.2	1.6	0.6	1.0	ns	11.6	0.3	0.5	1.0	ns
T × C	12	0.1	0.004	0.7	0.7	ns	25.9	2.2	0.8	0.7	ns	13.3	1.1	1.8	0.04	*
DAS × T × C	120	0.3	0.002	0.5	1.0	ns	173.7	1.5	0.5	1.0	ns	43.8	0.4	0.6	1.0	ns
Residuals	880	4.5	0.01				2419.5	2.8				541.7	0.6			

*, and ***, indicate significance levels at *p* < 0.05, and *p* < 0.001, respectively, and ns indicates non-significance. DAS-Days after stress, T-Treatment, C-Cultivar.

**Table 2:** F-statistics from a linear mixed effects model analysis of variance of the physiological traits, including stomatal conductance, transpiration, and leaf temperature difference measured in the field study from five peanut genotypes across two treatments.

Traits		Stomatal Conductance					Transpiration					Leaf Temperature Difference				
SOV	Df	Sum Sq	Mean Sq	F Value	Pr (>F)		Sum Sq	Mean Sq	F Value	Pr (>F)		Sum Sq	Mean Sq	F Value	Pr (>F)	
WAS	7	29.4	4.2	51.6	<2.2 × 10^−16^	***	3895.9	556.6	75.4	<2.2 × 10^−16^	***	268.3	38.3	21.8	<2.2 × 10^−16^	***
T	1	3.2	3.2	39.1	8.3 × 10^−10^	***	584.1	584.1	79.2	<2.2 × 10^−16^	***	0.2	0.2	0.1	0.7	ns
C	4	1.3	0.3	4.1	0.003	**	72.6	18.2	2.4	0.045	*	24.6	6.2	3.5	0.008	**
WAS × T	7	7.8	1.1	13.7	2.5 × 10^−16^	***	462.0	66.0	8.9	2.0 × 10^−10^	***	60.2	8.6	4.9	2.3 × 10^−05^	***
WAS × C	28	6.3	0.2	2.7	5.3 × 10^−06^	***	208.9	7.5	1.0	0.5	ns	43.6	1.6	0.9	0.6	ns
T × C	4	0.2	0.04	0.5	0.7	ns	9	2.3	0.3	0.9	ns	2.6	0.7	0.4	0.8	ns
WAS × T × C	28	3.0	0.1	1.3	0.1	ns	108.5	3.9	0.5	1.0	ns	37.2	1.3	0.8	0.8	ns
Residuals	520	42.6	0.08				3848.2	7.4				996.6	1.9			

*, **, and ***, indicate significance levels at *p* < 0.05, *p* < 0.01, and *p* < 0.001, respectively, and ns indicates nonsignificant. WAS-Weeks after stress, T-Treatment, C-Cultivar.

## Data Availability

Data are contained within the article and Supplementary Materials.

## Supplementary Materials

The following supporting information can be downloaded at: https://www.mdpi.com/article/10.3390/plants14172687/s1, Table S1. Microclimate maintained in the field study across treatments during the stress period; Figure S1: Growth chamber study representing treatments 50 days after stress. (A) Control (CNT), (B) drought stress (DS), (C) heat stress (HS), and (D) heat and drought stress (HS+DS).; Figure S2: Aerial view of plots from field-based study. (A) Combined heat and drought stress plots, and (B) Rainfed plots. The image was collected 70 days after stress.
