# Supplementary material for: Evaluation of Peanut Physiological Responses to Heat and Drought Stress Across Growth Chamber and Field Environments
Source: Plants (Basel). 2025 Aug 28;14(17):2687. doi: 10.3390/plants14172687 (PMC12430426; doi:10.3390/plants14172687)
Supplement: Supplementary file 1 [file plants-14-02687-s001.zip › plants-3813014-supplementary.pdf]

Supplementary Table S1. Microclimate maintained in the field study across treatments during the stress period.

**Microclimate**	**Rainfed/Control**	**Combined heat and drought stress**
Rainfall	303 mm	0 mm
Average soil moisture (Volumetric water content)	0.18 m^3 m^{-3}	0.06 m^3 m^{-3}
Average air temperature	24 °C	27 °C
Maximum air temperature	30 °C	39 °C
Average soil temperature	25 °C	26 °C
Maximum soil temperature	26 °C	27 °C

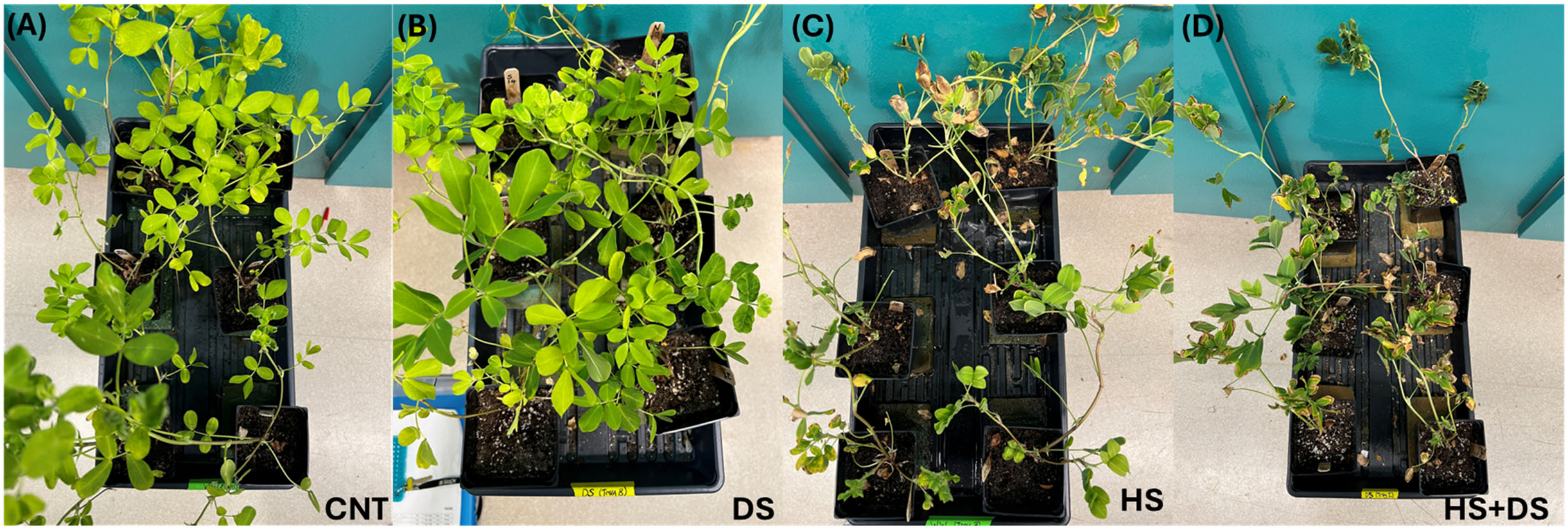

Supplementary Figure S1. Growth chamber study representing treatments 50 days after stress. (A) Control (CNT), (B) drought stress (DS), (C) heat stress (HS), and (D) heat and drought stress (HS+DS).

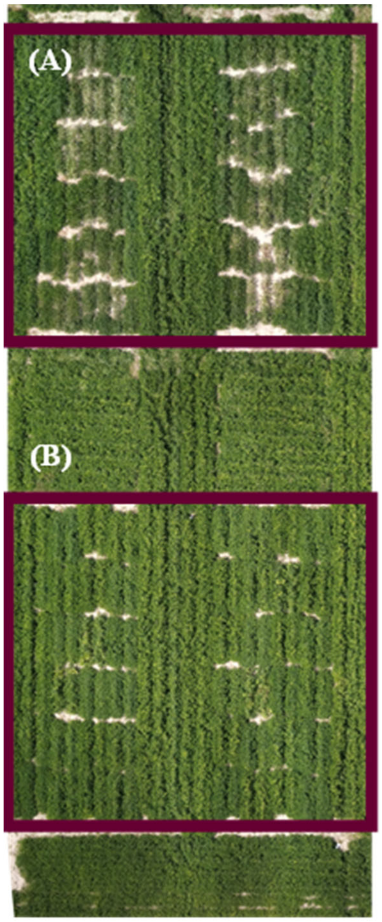

Supplementary Figure S2. Aerial view of plots from field-based study. (A) Combined heat and drought stress plots, and (B) Rainfed plots. The image was collected 70 days after stress.
